# Materia Medica, or Pharmacology and Therapeutics

**Published:** 1856-12

**Authors:** 


*Materia Medica, or Pharmacology and Therapeutics.* By Wm. Tully, M.D. Vol. I. No. 20, June, 1856. Springfield, Mass. published by Jefferson Church, M.D. 1856.

This interesting and original publication continues to be issued in numbers, and to maintain fully the character for merit which was exhibited in the first numbers.

Price, twenty-five cents per number.

